# Perceived Contributors to Job Quality and Retention at Home Care Cooperatives

**DOI:** 10.1001/jamanetworkopen.2025.4457

**Published:** 2025-04-07

**Authors:** Geoffrey M. Gusoff, Miguel A. Cuevas, Catherine Sarkisian, Madeline R. Sterling, Ariel C. Avgar, Gery W. Ryan

**Affiliations:** 1 Department of Family Medicine, David Geffen School of Medicine at the University of California, Los Angeles; 2 Division of General Internal Medicine and Health Services Research, David Geffen School of Medicine at University of California, Los Angeles; 3 Geriatric Research Education and Clinical Center, Veterans Affairs Greater Los Angeles Healthcare System, Los Angeles, California; 4 Department of Medicine, Weill Cornell Medicine, New York, New York; 5 School of Industrial and Labor Relations, Cornell University, Ithaca, New York; 6 Department of Health Systems Science, Kaiser Permanente Bernard J. Tyson School of Medicine, Pasadena, California

## Abstract

**Importance:**

To safely age at home, millions of older adults rely on the services of home care workers (HCWs), a workforce marked by poor working conditions, high turnover, and critical worker shortages. Home care cooperatives—businesses co-owned and controlled by HCWs—have demonstrated significantly lower turnover and higher job quality than traditional home care businesses, but the factors associated with these outcomes have not been investigated.

**Objective:**

To identify potential factors associated with higher job quality and lower turnover at home care cooperatives.

**Design, Setting, and Participants:**

In this qualitative study, semistructured interviews were conducted in English from November 2023 to June 2024. To be eligible for the study, participants had to be 18 years old or older, speak English, and currently be employed by a home care cooperative. Interviews were conducted remotely over Zoom with individual HCWs and staff from home care cooperatives from across the US. Data were analyzed from May to July 2024.

**Exposure:**

Employment at a home care cooperative.

**Main Outcomes and Measures:**

Interviews were audio recorded and transcribed. A thematic analysis approach was used to code interviews and identify major themes and subthemes, informed by a conceptual model of direct care worker job outcomes.

**Results:**

A total of 23 HCWs and 9 staff members (14 participants aged 20-39 years [44%]; 8 [25%] African American, 4 [13%] Latinx, and 17 [53%] non-Latinx White) from 5 home care cooperatives participated in the study. Participants identified 4 key aspects of the cooperative work environment they perceived to be contributing to higher job quality and lower turnover compared with traditional agencies: (1) greater workplace control regarding patient care, case assignments, and organizational policies; (2) stronger community support and camaraderie; (3) a deeper culture of respect in which HCWs felt more valued; and (4) higher overall compensation in the form of wages, benefits, or profit sharing.

**Conclusions and Relevance:**

In this qualitative study of HCWs and staff at home care cooperatives, participants perceived cooperatives’ relatively high levels of HCW control, community, respect, and compensation to be important contributors to HCWs’ retention in the field. The participatory structure and practices of home care cooperatives in these areas may represent novel approaches for home care agencies to reduce HCW turnover and improve job quality to address critical workforce shortages.

## Key Points

**Question:**

What are potential factors associated with home care workers’ (HCWs) significantly lower turnover and higher job quality when employed by home care cooperatives as compared with traditional agencies?

**Findings:**

In this qualitative study of 32 HCWs and staff from 5 home care cooperatives across diverse settings, participants described 4 factors they perceived to contribute to relatively low turnover and high job quality at cooperatives: (1) HCW control over patient care, client assignments, and workplace policies; (2) community support; (3) respect; and (4) overall compensation.

**Meaning:**

These findings suggest that home care cooperatives’ participatory structure and HCW-centered practices may represent important approaches to attract and retain HCWs and address current workforce shortages.

## Teaser

This qualitative study examines health care workers' perspectives on job quality and turnover at home care cooperatives.

## Introduction

The US is in the midst of a growing crisis in home care. The vast majority of older adults seek to remain in their homes as they age, which often requires home care workers (HCWs) to provide critical assistance with activities of daily living.^1,2^ Although demand for HCWs is at an all-time high—with an additional 800 000 HCWs needed over the next 10 years—the relative supply of HCWs is at a historic low—with the per capita number of HCWs declining in recent years.^3,4^ These historic shortages are compounded by HCW turnover rates of up to 82% per year.^5^ The resulting decline in access to consistent home care places millions of older adults at increased risk for costly and avoidable hospital and nursing home stays, while increasing strain on millions of family caregivers.^6,7,8,9^

Studies suggest this HCW workforce crisis is largely driven by low job quality, marked by poor working conditions, limited benefits, and the lowest wages in health care.^10,11,12,13,14,15,16^ In addition, HCWs at traditional home care businesses often report they are disrespected and their input is ignored, despite substantial evidence that participation in workplace decisions is associated with higher job satisfaction and retention.^17,18,19,20,21,22,23^

In this HCW landscape of low-quality, high-turnover jobs, home care cooperatives—businesses co-owned and controlled by HCWs themselves—have emerged as positive outliers. At cooperatives, HCWs can become members (co-owners) after a probationary period, which allows them to vote for board members and on major decisions, serve on the board, and share in agency profits, providing unique levels of workplace participation.^24,25,26^

Since the formation of the first US home care cooperative in 1985, the model has grown to include over 14 cooperatives and 2050 HCWs, which have demonstrated half the turnover rates of comparable home care agencies.^27^ If home care cooperatives’ levels of HCW retention were achieved industry-wide, it would lead to savings of approximately $2.4 billion in direct turnover costs annually.^28,29,30^

While agency-level studies and industry analyses have consistently found significantly higher HCW job quality and lower turnover at home care cooperatives, the specific factors associated with these outcomes across cooperatives have not been rigorously examined.^25,27,29,31,32^ A better understanding of factors that may contribute to cooperatives’ exceptional HCW job quality and retention could generate testable hypotheses for subsequent studies and ultimately inform industry-wide efforts to attract and retain HCWs and address the workforce crisis in home care. Therefore, we conducted a qualitative study of the experiences of HCWs and other cooperative staff to identify and categorize factors they perceived as contributing to higher HCW job quality and lower turnover at home care cooperatives.

## Methods

### Setting and Study Population

The study was conducted from November 2023 to June 2024 with employees from home care cooperatives across the US. Participants were recruited in collaboration with ICA Group, a nonprofit supporting home care cooperative development nationally.

To obtain a diverse sample of individuals and cooperatives, we employed a 2-level sampling strategy. First, we recruited cooperatives of various agency sizes, tenures, primary payers, and geographies. Then, we recruited HCWs and staff within each cooperative, aiming for variation across role and tenure. We included office-based staff (ie, schedulers, managers), who were often former HCWs, given their additional perspectives on the impacts of cooperatives’ practices and cultures across HCWs. To meet eligibility criteria, participants had to be 18 years old or older, speak English, and currently be employed by a home care cooperative. The study focused on English speakers since all but 1 of the cooperatives from the diversity sample were exclusively or nearly exclusively composed of English speakers.

The principal investigator (G.M.G.) emailed a standardized script to home care cooperative contacts provided by ICA Group. Participating cooperatives provided lists of employees, who were emailed a standardized script to describe the study and evaluate their eligibility. Eligible participants were invited to an interview.

All participants provided informed verbal consent and received a $50 gift card. The study used Consolidated Criteria for Reporting Qualitative Research (COREQ) for the reporting of study design, methods, and outcomes. The institutional review board at the University of California, Los Angeles approved the study protocol.

### Data Collection

The principal investigator (G.M.G.) used a semistructured interview guide (eAppendix in Supplement 1) to conduct 45- to 60-minute interviews over Zoom. The interview guide was informed by a conceptual model from Zarska et al^13^ on the impact of working conditions on direct care worker outcomes and input from HCWs, administrators, and other key informants.

To identify perceived job quality and turnover-related factors at cooperatives, participants were asked about their overall experience at the cooperative, HCW workplace culture, participation in decision-making, and how these compared with experiences at other home care agencies. They were asked what aspects of the cooperative, if any, they thought contributed to HCW job satisfaction and retention. Participants were also asked about factors at cooperatives they perceived as contributing to care quality, which were analyzed in a separate analysis. Participants provided employment and demographic information, including their tenure at the cooperative, years of HCW experience, age, as well as race and ethnicity (African American, Latinx, and non-Latinx White), given the role of racialization in HCW workforce organization and outcomes.^11,33^

### Data Analysis

Interviews were audio recorded and transcribed. Transcripts were coded by 2 investigators (G.M.G. and M.A.C.) using Dedoose software version 9.2.7 (Dedoose) under the supervision of a qualitative methods expert (G.W.R.). We employed a thematic analysis coding approach as described by Ryan and Bernard, combining deductive and inductive methods.^34,35,36^ First, we applied a small set of broad codes informed by the conceptual model and key informants. Then, the coding investigators met regularly to compare coding, resolve discrepancies through consensus, refine codes as needed, and identify subthemes. Themes and subthemes were assessed for commonality, salience, and variation across individuals and work settings.

## Results

### Participant Characteristics

A total of 32 participants employed across 5 cooperatives participated in the study, including 23 HCWs and 9 staff members (14 participants aged 20-39 years [44%]; 8 [25%] African American, 4 [13%] Latinx, and 17 [53%] non-Latinx White). Cooperatives varied by size, tenure, primary payer, and geography, with 2 in large urban areas, 2 in small urban and suburban areas, and 1 in a rural area. Participants were diverse in age, racial and ethnic background, and experience, with 20 participants (63%) reporting experience working as caregivers outside the cooperative. Table 1 and Table 2 summarize the characteristics of the participating cooperatives and individuals.

**Table 1.** Characteristics of Participating Cooperatives

Characteristics	Cooperatives, No. (%) (N = 5)
Cooperative size	
Small (<50 employees)	2 (40)
Medium or large (≥50 employees)	3 (60)
Primary payer	
Medicaid	3 (60)
Private pay	2 (40)
Market density	
Large urban	2 (40)
Small urban/suburban	2 (40)
Rural	1 (20)
Region	
Northeast	2 (40)
Midwest	1 (20)
Northwest	2 (40)
Agency tenure, y	
>15	3 (60)
7-15	1 (20)
0-6	1 (20)

**Table 2.** Characteristics of Participating Individuals

Characteristic	Participants, No. (%) (N = 32)
Role	
Home care worker	23 (72)
Staff	9 (28)
Worker tenure, y	
<2	13 (41)
2-5	8 (25)
6-10	5 (16)
>10	6 (19)
Membership status	
Worker-owner	29 (91)
Nonworker-owner	3 (9)
Race and ethnicity	
African American	8 (25)
Latinx	4 (13)
Non-Latinx White	17 (53)
2 or more races	2 (6)
Declined	1 (3)
Age, y	
20-29	7 (22)
30-39	7 (22)
40-49	6 (19)
50-59	3 (9)
60-69	7 (22)
≥70	2 (6)
Cooperative board involvement	
Current member	8 (25)
Former member	6 (19)
Never member	18 (56)
Other paid caregiving experience	
Yes	20 (63)
No	12 (38)

### Major Themes

Four major themes emerged from the interviews: control, community, culture of respect, and compensation. Descriptions of each theme and a list of subthemes are included in Table 3.

**Table 3.** Interview Themes and Subthemes

Theme	Description	Subthemes
Control	Level of say, input, or voice that home care workers have in various aspects of their work	Input in patient care, input in scheduling and client matching, and input in agency policies
Community support	Experience of community, belonging, or teamwork at the home care cooperative	Support from staff and colleagues and sense of belonging and connection
Culture of respect	Experience of being respected, valued, or recognized in the workplace	Informal interactions and formal recognition
Compensation	Wages, benefits, or other compensation received by home care workers as part of the home care cooperative	Wages, benefits, and other compensation (eg, profit sharing)

#### Theme 1: Control

The most common theme discussed by participants was the relatively high levels of HCW input and control at cooperatives, especially compared with other agencies. They described this input and control in 3 domains: patient care, scheduling and patient assignments, and agency-wide policies.

While participants noted that care is largely dictated by the patient’s care plan, many also described significant autonomy and input into the care plan. One HCW shared, “Whether it’s changing the care plan or just how I do things in general, I have a pretty big say.” In contrast, an HCW described her previous agency’s response to her care input, recalling, “They just blew it off and they didn’t care.”

Participants also described control over work schedules and assignments as important for HCW experience and retention at the cooperatives. One HCW noted, “The office is really willing to work with you, they’ll give you as many or as little hours as you want…so yeah, I don’t see myself straying far from [the cooperative].” While assignments are still constrained by patients’ availabilities and preferences, another HCW observed, “I do have a lot more say than I would in another setting that’s not a co-op.”

Finally, HCWs emphasized the control they had over organizational policies and decisions, often exercised through HCW-elected boards, committees, and member-wide votes. One HCW noted, “You’re part of how the company is run, your opinion counts.” HCWs’ depth of participation in decision-making varied significantly based on personal interest, schedule availability, and other factors. HCWs who served as board members described that experience as providing a sense of “accomplishment” and “motivation.” Conversely, one HCW linked low control to higher turnover at traditional agencies, explaining, “If a bunch of people disagree with the boss but they don’t change their ways, instead of talking it through because they’re both owners or they’re part of a team, those people might just leave.”

#### Theme 2: Community Support

While acknowledging isolating aspects of providing in-home care, participants also frequently described cooperatives’ “sense of community,” “camaraderie culture,” and “sense of teamwork.” HCWs noted this sense of community derived in part from “the fact that we are part owners.”

One important aspect of community at the cooperatives was support from staff and other HCWs. One HCW said of the cooperative’s staff, “They’re always there, they’re always available.” Another noted, “If I’m having a really bad day, I know I can pick up the phone, and I can call [the coordinator], and I can vent for 10 minutes.” Of fellow HCWs, one participant noted, “You get to know people at the co-op, and you know that you can rely on them if you need somebody to talk to.” One larger cooperative institutionalized this HCW support through a peer mentor program while others encouraged community through events like barbecues, parades, and in-person membership meetings.

A related aspect of community described by participants was a “sense of family,” “personal connection,” and “belonging,” despite limited in-person contact with colleagues. One HCW noted this sense of community “Makes me want to stay at [the cooperative] longer…it makes me feel like I’m part of something.” Another HCW described how HCWs’ needs are not ignored or forgotten at cooperatives, reflecting, “No one’s going to slip through the cracks in a co-op, whereas in a corporate setting it happens all the time, every day, by the hour.”

#### Theme 3: Culture of Respect

Several participants also noted a unique culture of respect for HCWs at the cooperatives. HCWs described “just being treated like a person,” and enjoying that “everyone treats each other with respect,” and “[feeling] way more valued here.” They contrasted this to other agencies where 1 participant noted, “I don’t recall feeling that I was cared about as much,” and “They don’t consider the employee, they only consider the dollars.” Participants attributed cooperatives’ culture of respect to various factors, including the “equal playing field” of cooperatives’ 1-member 1-vote model, the central role of HCW input, or the fact that many staff have been HCWs themselves.

Larger cooperatives reinforced this culture of respect through formal events and committees, including Caregiver Appreciation Day or a Happiness Committee celebrating HCWs’ birthdays. While disrespect from patients or staff was not completely absent at the cooperatives, participants consistently reported the culture was notably better than what they experienced at other agencies. Regarding turnover, one HCW noted, “As far as keeping your employees, recognition goes a long way.”

#### Theme 4: Compensation

Many participants noted that cooperatives provided better overall compensation—including wages, benefits, and profit sharing—than other agencies. Participants attributed this to HCWs’ role as co-owners and key decision-makers in setting compensation policy. As 1 HCW noted, “We’re probably more likely to get raises more frequently because we’re voting on it for ourselves.”

The form that compensation took varied across cooperatives. Smaller cooperatives, lacking the scale to offer affordable group-level benefits like health insurance, focused on providing higher wages and profit sharing, with 1 staff member noting, “We are definitely, if not the highest, 1 of the highest paying agencies in [the region].” Another smaller cooperative provided profit sharing of up to $3400 for each HCW.

In contrast, larger cooperatives often prioritized health insurance and other benefits valued by HCWs. A staff member noted, “You can go to another agency that pays 2 more dollars than what we pay, or like $22 an hour, but you’re not going to get…the same level of benefits.” An HCW at another larger cooperative recalled, “When I got to [the cooperative], I never had the benefits like we had.”

Across cooperatives, this emphasis on HCW compensation was thought to play an important role in HCW retention. An HCW at a smaller cooperative stated, “The pay is competitive, so I don’t have any financial reason to look elsewhere.” One staff member from a larger cooperative noted, “A lot of [HCWs] say, ‘I have to think twice about leaving because I don’t want to leave these benefits behind.’”

### Variations Across Themes

In addition to variations among participant responses within each theme, there were also variations across themes. Staff, HCWs with longer tenure, and HCWs with board experience were somewhat more likely to specifically attribute workplace conditions to the cooperative model. Participants from smaller cooperatives tended to emphasize informal aspects of workplace culture (eg, interpersonal relationships) while those from larger cooperatives spoke more about formal aspects (eg, special committees or roles). Otherwise, we did not observe consistent differences in responses based on respondent role or other individual or agency characteristics.

## Discussion

In this qualitative study, participants described greater control, community support, respect, and compensation as key features of cooperatives’ work environments and important contributors to higher job quality and retention. The importance of each of these factors for workforce outcomes is supported by prior studies, but the specific experience of cooperative participants offers novel insights.

First, while worker control and community support have been identified as fundamental factors for reducing job strain and turnover, these factors are inconsistently defined in the HCW literature and are often overlooked as areas for intervention.^10,12,17,18,19,37,38,39,40,41,42^ Our findings suggest a 3-level approach to HCW control—regarding patient care, case matching, and agency policies—may help capture distinct aspects of HCW input and autonomy. Similarly, our findings suggest assistance with job tasks and feelings of belonging are both important components of community support for HCWs. These multifaceted, HCW-informed conceptualizations of control and community support can guide future research and inform potential interventions, such as HCW board participation or peer mentoring programs.

Second, participants perceived strong cultures of respect to be an important contributor to job quality and retention at cooperatives, consistent with broader findings among HCWs.^11,43,44^ While a culture of respect is often treated as a nebulous quality rooted in individual personalities, participants often described it as the result of organizational structures and practices, such as HCWs’ status as co-owners and key decision-makers and employing staff who were formerly HCWs. This suggests potential interventions for enhancing agencies’ cultures of respect.

Finally, HCW turnover has been consistently associated with low compensation, particularly low hourly wages.^10,15,45,46^ Our findings affirm the critical role of competitive wages in retention but also emphasize the importance of other forms of compensation, including health insurance and paid time off. Notably, in the cooperatives studied, where HCWs play a large role in setting compensation policy, HCWs invested in these benefits when possible rather than simply maximizing wages. This suggests efforts to improve HCW job quality and retention should focus not only on higher wages but also on increasing benefits like health insurance and paid time off.

Our findings suggest low job quality and high turnover are not intrinsic features of the HCW industry but rather the results of modifiable practices, structures, and policies.^44^ Practices that increase HCW control, community, respect, and compensation may significantly improve HCW job quality and reduce turnover. Consistent with the literature on high-performance work systems, participants described these practices at cooperatives as deeply interrelated and synergistic, with shared decision-making informing compensation policy, facilitating community-building, and supporting a culture of respect while greater compensation, community, and respect facilitate increased HCW participation.^47,48,49^ As a result, focusing only on a single factor—eg, HCW appreciation events—may have much less impact on retention than addressing multiple factors simultaneously.

At the cooperatives, these job-enhancing practices were not just described as synergistic but also as structurally embedded. Participants described how the cooperative structure, in which HCWs are co-owners and key decision-makers, contributed to differences in control, community, respect, and compensation. While additional studies are needed to evaluate this potential causal relationship, these observations suggest that structural arrangements—eg, HCW profit-sharing, board participation, and even co-ownership—may facilitate the adoption of practices that improve job quality and reduce turnover.^25,26,50^

Addressing the perceived contributors to HCW job quality and turnover at the scale needed to combat the current crisis also requires industry-wide policy reforms. These may include increased public investments in home care (such as that proposed in the Better Care Better Jobs Act), improved integration of HCWs into the care team, expanded HCW unionization (which can facilitate HCW group benefits and policy advocacy among other benefits), and business development support to promote the expansion of home care cooperatives.^51,52,53,54,55^ By promoting a consistent and growing HCW workforce, these types of agency-level changes and industry-wide reforms have the potential to significantly improve the lives of millions of older adults, family caregivers, and HCWs while preventing costly, unnecessary care.^6,7,9^

### Limitations

This study has several limitations. Including only cooperative employees provided a more in-depth exploration of cooperatives but precluded a direct comparison with other agency types. While most participants had experience at other agencies for comparison, these comparisons may be subject to recall bias and/or selection bias. In addition, non-English–speaking HCWs may have different experiences of cooperative and noncooperative workplaces not captured in this analysis. Additionally, while participants attributed certain workplace characteristics to the cooperative model itself, it is possible that other factors (eg, agency size) contributed significantly to those characteristics. The persistence of themes across size and payer type in the sample suggests the cooperative structure itself may be associated with these characteristics, but a follow-up study comparing conditions at cooperatives and noncooperatives, including speakers of other languages, and controlling for these types of covariates could further elucidate the cooperative difference in home care.

## Conclusions

In this qualitative study of home care cooperative HCWs and staff, participants perceived relatively high levels of HCW control, community, respect, and compensation to be key contributors to cooperatives’ higher HCW job quality and retention. These initial findings suggest cooperatives’ practices in these areas, as well as agency structures that increase HCW ownership and participation in decision-making, may represent effective approaches to addressing the HCW workforce crisis. However, additional studies are needed to further elucidate the role of each factor and their applicability across contexts.

## Notes

Supplement 1. **eAppendix.** Interview Guide

Supplement 2.
Data Sharing Statement
