# Supplementary material for: Perceived Contributors to Job Quality and Retention at Home Care Cooperatives
Source: JAMA Netw Open. 2025 Apr 7;8(4):e254457. doi: 10.1001/jamanetworkopen.2025.4457 (PMC11976488; doi:10.1001/jamanetworkopen.2025.4457)
Supplement: Supplement 1. — eAppendix. Interview Guide [file jamanetwopen-e254457-s001.pdf]

Supplemental Online Content

Gusoff GM, Cuevas MA, Sarkisian C, Sterling MR, Avgar AC, Ryan GW. Perceived factors associated with higher job quality and lower turnover at home care cooperatives. *JAMA Netw Open*. 2025;8(4):e254457. doi:10.1001/jamanetworkopen.2025.4457

eAppendix . Interview Guide

This supplemental material has been provided by the authors to give readers additional information about their work.

© 2025 Gusoff GM et al. *JAMA Network Open*.

eAppendix. Interview Guide

Semi-structured interview prompts are provided below. The interview is designed to last 45 to 60 minutes and to be conducted over Zoom. The interview structure is organized to first provide general information and obtain consent, then to elicit background and demographic information from participants, followed by "Grand Tour" open ended questions to describe general experiences working at a home care cooperative and impressions about the impact of the cooperative model, and finally to explore specific aspects of the cooperative work environment, their perceived impacts on HCW job quality, care quality, and turnover, and how they compare to other caregiving environments where participants have worked (if applicable).

NOTE: This guide provides questions and follow-up probes to cover various aspects of participant experiences and perspectives. Questions were not always asked in order or with the same follow-up probes, and not all questions were asked to all participants, as many participants covered later areas in their response to earlier questions (e.g. Grand Tour questions).

Reminder of Purpose, Confidentiality, and Ability to End Interview

Thanks for agreeing to participate in this interview. The goal of these interviews is to better understand how different types of home care agencies, like home care cooperatives, can affect the lives of home health aides and the clients they care for. We're interviewing home health aides and other staff from several home care agencies across the country to learn from your perspectives. We hope to identify ways to improve how home care businesses are set up to make life better for you and the clients you care for.

In the next 45 minutes to an hour, I will ask you some questions to help me understand how your experiences working with [*cooperative*] have impacted you as a caregiver, employee, and as a person. I'm going to ask you about what it's really like to work here, the good and the bad, what's working and what's not working. We want to remind you that your participation in this conversation is voluntary and there is no penalty if you decline or choose not to answer any of the questions. In appreciation for your time and insights, I'll be sending you a $50 Visa gift card by email after the interview.

We hope to share what we learn from these interviews, but your specific answers are confidential and will not be shared with [*cooperative*]. These interviews will be recorded so I can reference things you've said. Anything we share will be de-identified, meaning nothing you say will be connected to you or your name. In fact, I will not even write your name down in my notes. The notes and recording of this conversation will be stored in a password-protected, secured location and once we have finished the analysis, the notes and recordings will be destroyed. You can contact me at the phone number or email address provided in my outreach email if you have any follow up concerns or questions.

Now that we've reviewed this information, do you agree to go ahead with the recorded interview?

Do you have any questions before we begin?

© 2025 Gusoff GM et al. *JAMA Network Open*.

Background Questions
To start off, I want to ask a few questions to get a sense of your background and role at [cooperative] to get a sense of where you're coming from.

What is your job at [*cooperative*]?
How many years have you worked at [*cooperative*]?
About how many hours a week do you work as an aide with [*cooperative*]?
Have you worked as a caregiver with other home care businesses?
Are you a worker-owner/member of [*cooperative*]?
Are you part of the cooperative board or any committees?
Do you have any other leadership role at [*cooperative*]?
Home health aides come from many different backgrounds in terms of their race/ethnicity, age, and other characteristics. How do you identify in terms of your race/ethnicity?
Would you mind sharing your approximate age, if you're in your 20s, 30s, 40s, etc.?

Grand Tour Questions
As a primary care doctor, I see lots of patients in the office, but I don't know at all what it's like being a home health aide and caring for people in their homes.

Can you take a minute and describe what it's like to work for [*cooperative*] for someone who has never worked there?

I'm really interested in your own journey. Can you tell me what brought you to [*cooperative*]?
What keeps you at [*cooperative*]?
Where do you see yourself going in the future?

What does it mean to you that you work at a worker-owned cooperative?
What benefits, if any, do you see to being part of a cooperative?

What downsides, if any, do you see from being part of a cooperative?

Care Quality

As a primary care doctor, I'm not always able to provide the level of care I wish I could due to all sorts of factors. On a scale of 1 to 10, 10 being the best possible care and 1 being the worst care, how would you rate the care you're able to provide to your clients?

Why did you choose that number?
What would it take to make that number higher?
How does that number compare to other places you've worked as an aide?

Some people have said the cooperative (/being a worker owner) makes the care you provide different. What are your thoughts on that?

© 2025 Gusoff GM et al. *JAMA Network Open*.

Organizational Aspects
So we've talked about clients and what happens in the home. I want to switch gears and see if you can help me understand how [*cooperative*] works.

Let's start with you. What's your role in the cooperative?

Can you walk me through a typical day working at [*cooperative*]?

What other people at the cooperative do you work with to get your work done? How do you interact with them?
[Schedulers, nurses, managers, etc.]

Is there anyone else at the cooperative who impacts (helps or gets in the way of) getting your job done?

How does that number compare to other places you've worked as an aide?

Workplace Participation

I'm interested in how much say you have in your work, by which I mean how much your opinion or perspective is taken into account in different situations.

First I want to ask how much say you have in how you take care of your clients, on a scale of 1 to 10, with 10 being you have a lot of say and others always take your opinions into account and 1 is when you have no say and people always ignore your input.
Why did you choose that number?
Do you think that number is the same or different for other aides at [*cooperative*]?
Can you describe for me ways, if any, you have a say in client care?
How do you think having a say in caring for your clients impacts your job?
How does that compare to other places you've worked as an aide?
To what extent, if any, do you think working at a cooperative impacts that number?

Now I want to ask how much say you have in day-to-day things at the cooperative like your schedule or the types of clients you get matched with. How much say do you have in these day-to-day issues on a scale of 1 to 10, with 10 being a lot of say and 1 being no say at all?
Why did you choose that number?
Do you think that number is the same or different for other aides at [*cooperative*]?
Can you describe for me ways, if any, you have a say in day-to-day decisions like scheduling?
How do you think having a say in these day-to-day issues impacts your job?
How does that compare to other places you've worked as an aide?
To what extent, if any, do you think working at a cooperative impacts that number?

Lastly, I want to ask how much say you have in the big decisions of the cooperative [like who gets hired and how extra money gets distributed]. How much say do you have in these big decisions on a scale of 1 to 10, with 10 being a lot of say and 1 being no say at all?

© 2025 Gusoff GM et al. *JAMA Network Open*.

Why did you choose that number?
Do you think that number is the same or different for other aides at [*cooperative*]?
Can you describe for me ways, if any, you have a say in big decisions?
How do you think having a say in big decisions impacts your job?
How does that compare to other places you've worked as an aide?
To what extent, if any, do you think working at a cooperative impacts that number?

Organizational Culture

On a scale of 1 to 10, to what extent do you feel like you really "own" the business, with 10 being that you really feel you own the business and 1 meaning you don't feel you own the business at all.

How do you think that impacts your job?
How does that compare to other places you've worked as an aide?
To what extent, if any, do you think working at a cooperative impacts that number?

On a scale of 1 to 10, to what extent do you feel a sense of belonging at the cooperative?
How do you think that impacts your job?
How does that compare to other places you've worked as an aide?
To what extent, if any, do you think working at a cooperative impacts that number?

Overall Job Quality/Satisfaction

People have different levels of how satisfied they are with their job. On a scale of 1 to 10, how satisfied are you with your job at [cooperative], 10 being "I absolutely love it" and 1 being "I absolutely hate it"?
Why did you choose that number?
What would it take to make that number higher?
How does that number compare to other places you've worked as an aide?
How do you think that compares to other aides at the cooperative?
How do you think working at a cooperative impacts that number?

Turnover/Retention

Being a home health aide can be a really hard job, and a lot of people end up leaving to work for another agency or a different job altogether.
What do you think are the most important things agencies can do to support aides so they don't end up leaving?
On a scale of 1 to 10, how often are these things happening at [*cooperative*] with 1 being "never" and 10 being "always".
Why did you choose that number?
How does that compare to other places you've worked?
Do you think being a cooperative impacts those things? If so, how?

© 2025 Gusoff GM et al. *JAMA Network Open*.

Home care cooperatives have half the rates of people leaving as home care businesses that aren't cooperatives. Why do you think that is?

<u>Sounding Board Questions</u>
[*NOTE: These questions were added in later interviews if time permitted and if not addressed elsewhere to assess participants' thoughts on themes identified in prior interviews. If asked, these questions were included at the end of the interview to avoid influencing other responses.*]

Material Benefits
How do the wages, benefits, and training at [*cooperative*] compare to other agencies in your area?
Some people have told me that because workers co-own the business at a cooperative, it means more of that money goes to benefit the workers [through higher wages, better benefits, or better training] compared to agencies where extra money goes to the owner? What do you think about that?

Culture/Community
Some people have told me the cooperative model leads to a different culture [with people feeling a stronger sense of community, support, and caring among their co-workers]. What do you think about that?
How do you think the culture at the cooperative impacts the aides? How does it impact the clients?

Participation
Some people have told me they like the coop model because they like being part of decisions that affect them and like having more say [in how they care for clients and how the business is run]. What do you think about that?

<u>Closing Questions</u>
What is something you wish people knew about what it's like to work at [cooperative], positive or negative, that we haven't talked about yet?

Is there anything else we haven't talked about that you'd like people to know about how working at [cooperative] has impacted your client care, your job, or your life in general?

<u>Closing Script</u>
This has been incredibly informative, and I really appreciate you taking out the time to discuss this with me. Thanks again for your time and insights.

© 2025 Gusoff GM et al. *JAMA Network Open*.

Interview Guide – Office-Based Staff Version

Semi-structured interview prompts are provided below. The interview is designed to last 45 to 60 minutes and to be conducted over Zoom. The interview structure is organized to first provide general information and obtain consent, then to elicit background and demographic information from participants, followed by "Grand Tour" open ended questions to describe general experiences of HCWs working at a home care cooperative and impressions about the impact of the cooperative model, and finally to explore specific aspects of the cooperative work environment, their perceived impacts on HCW job quality, care quality, and turnover, and how they compare to other caregiving environments where participants have worked (if applicable).

NOTE: This guide provides questions and follow-up probes to cover various aspects of participant experiences and perspectives. Questions were not always asked in order or with the same follow-up probes, and not all questions were asked to all participants, as many participants covered later areas in their response to earlier questions (e.g. Grand Tour questions).

Reminder of Purpose, Confidentiality, and Ability to End Interview

Thanks for agreeing to participate in this interview. The goal of these interviews is to better understand how different types of home care agencies, like home care cooperatives, can affect the lives of home health aides and personal care aides and the clients they care for. We're interviewing aides and other staff from several home care agencies across the country to learn from your perspectives. We hope to identify ways to improve how home care agencies are set up to make life better for aides and the clients they care for.

In the next 45 minutes to an hour, we will ask you some questions to help us understand how various aspects of [*cooperative*] have impacted the aides as caregivers, employees, and as people. I'm going to ask you about what it's really like to work at [*cooperative*], the good and the bad, what's working and what's not working. We want to remind you that your participation in this conversation is voluntary and there is no penalty if you decline or choose not to answer any of the questions. In appreciation for your time and insights, I'll be sending you a $50 Visa gift card by email after the interview.

We hope to share what we learn from these interviews, but your specific answers are confidential and will not be shared with others at your home care agency. These interviews will be recorded so I can reference things you've said. Anything we share will be de-identified, meaning nothing you say will be connected to you or your name. In fact, I will not even write your name down in my notes. The notes and recording of this conversation will be stored in a password-protected, secured location and once we have finished the analysis, the notes and recordings will be destroyed. You can contact me at the phone number or email address provided in my outreach email if you have any follow up concerns or questions.

Now that we've reviewed this information, do you agree to go ahead with the recorded interview?

Do you have any questions before we begin?

© 2025 Gusoff GM et al. *JAMA Network Open*.

Background Questions
To start off, I want to ask a few questions to get a sense of your background and role at [cooperative] to get a sense of where you're coming from.

What is your job at [*cooperative*]?
How many years have you worked at [*cooperative*]?
About how many hours a week do you work with [*cooperative*]?
Have you worked as a caregiver with other home care businesses?
Are you a worker-owner/member of [*cooperative*]?
Are you part of the cooperative board or any committees?
Do you have any other leadership role at [*cooperative*]?
People working in home care come from many different backgrounds in terms of their race/ethnicity, age, and other characteristics. How do you identify in terms of your race/ethnicity?
Would you mind sharing your approximate age, if you're in your 20s, 30s, 40s, etc.?

Grand Tour Questions
As a primary care doctor, I see lots of patients in the office, but I don't know at all what it's like being a home health aide and caring for people in their homes.

Can you take a minute and describe what it's like to work for [*cooperative*] for someone who has never worked there?

What do you think brings home health aides to work at [*cooperative*]?
What do you think keeps them working at [*cooperative*]?

What does it mean to you that you work at a worker-owned cooperative?
What benefits, if any, does the cooperative model provide for aides?
What downsides, if any, does the cooperative model have for aides?

Care Quality

As a primary care doctor, I'm not always able to provide the level of care I wish I could due to all sorts of factors. On a scale of 1 to 10, 10 being the best possible care and 1 being the worst care, how would you rate the care [*cooperative*] aides provide to your clients?

Why did you choose that number?
What would it take to make that number higher?
How does that number compare to other places you've worked?

Some people have said the cooperative model changes the way aides provide care. What are your thoughts on that?

© 2025 Gusoff GM et al. *JAMA Network Open*.

Organizational Aspects
So we've talked about clients and what happens in the home. I want to switch gears and see if you can help me understand how [*cooperative*] works.

Let's start with you. What's your role in the cooperative?

Can you walk me through a typical day working at [*cooperative*]?

In what ways do you interact with aides?

What other staff do aides interact with and in what ways?

Workplace Participation

I'm interested in how much say aides have in their work, by which I mean how much their opinion or perspective is taken into account in different situations.

First I want to ask how much say aides have in how they take care of their clients, on a scale of 1 to 10, with 10 being you have a lot of say and others always take their opinions into account and 1 is when they have no say and people always ignore their input.
Why did you choose that number?
Do think that number varies depending on the aide?
Can you describe for me ways, if any, they have a say in client care?
How do you think having a say in caring for their clients impacts their job?
How does that compare to other places you've worked?
To what extent, if any, do you think working at a cooperative impacts that number?

Now I want to ask how much say aides have in day-to-day things at the cooperative like their schedule or the types of clients they get matched with. How much say do aides have in these day-to-day issues on a scale of 1 to 10, with 10 being a lot of say and 1 being no say at all?
Why did you choose that number?
Do think that number varies depending on the aide?
Can you describe for me ways, if any, aides have a say in day-to-day decisions like scheduling?
How do you think having a say in these day-to-day issues impacts their job?
How does that compare to other places you've worked?
To what extent, if any, do you think working at a cooperative impacts that number?

Lastly, I want to ask how much say aides have in the big decisions of the cooperative [like who gets hired and how extra money gets distributed]. How much say do aides have in these big decisions on a scale of 1 to 10, with 10 being a lot of say and 1 being no say at all?
Why did you choose that number?
Do think that number varies depending on the aide?
Can you describe for me ways, if any, aides have a say in big decisions?
How do you think having a say in big decisions impacts their job?
How does that compare to other places you've worked?

© 2025 Gusoff GM et al. *JAMA Network Open*.

To what extent, if any, do you think working at a cooperative impacts that number?

Organizational Culture
On a scale of 1 to 10, to what extent do you think aides feel like they really "own" the business, with 10 being that they really feel they own the business and 1 meaning they don't feel they own the business at all.

How do you think that impacts their job?
How does that compare to other places you've worked as an aide?
To what extent, if any, do you think working at a cooperative impacts that number?

On a scale of 1 to 10, to what extent do you think aides feel a sense of belonging at the cooperative?
How do you think that impacts their job?
How does that compare to other places you've worked as an aide?
To what extent, if any, do you think working at a cooperative impacts that number?

Overall Job Quality/Satisfaction
People have different levels of how satisfied they are with their job. On a scale of 1 to 10, how satisfied do you think aides are with their job at [*cooperative*], 10 being "They absolutely love it" and 1 being "They absolutely hate it"?
Why did you choose that number?
What do you think it would take to make that number higher?
How does that number compare to other places you've worked?
How do you think that number varies across different aides?
How do you think working at a cooperative impacts that number?

Turnover/Retention
Being a home health aide can be a really hard job, and a lot of people end up leaving to work for another agency or a different job altogether.
What do you think are the most important things agencies can do to support aides so they don't end up leaving?
On a scale of 1 to 10, how often are these things happening at [*cooperative*] with 1 being "never" and 10 being "always".
Why did you choose that number?
How does that compare to other places you've worked?
Do you think being a cooperative impacts those things? If so, how?

Home care cooperatives have half the rates of people leaving as home care businesses that aren't cooperatives. Why do you think that is?

© 2025 Gusoff GM et al. *JAMA Network Open*.

Sounding Board Questions

[*NOTE: These questions were added in later interviews if time permitted and if not addressed elsewhere to assess participants' thoughts on themes identified in prior interviews. If asked, these questions were included at the end of the interview to avoid influencing other responses.*]

Material Benefits

How do the wages, benefits, and training at [*cooperative*] compare to other agencies in your area?

Some people have told me that because workers co-own the business at a cooperative, it means more of that money goes to benefit the aides [through higher wages, better benefits, or better training] compared to agencies where extra money goes to the owner? What do you think about that?

Culture/Community

Some people have told me the cooperative model leads to a different culture for aides [with people feeling a stronger sense of community, support, and caring among their co-workers]. What do you think about that?

How do you think the culture at the cooperative impacts the aides? How does it impact the clients?

Participation

Some aides have told me they like the coop model because they like being part of decisions that affect them and like having more say [in how they care for clients and how the business is run]. What do you think about that?

Closing Questions

What is something you wish people knew about what it's like to work at [cooperative], positive or negative, that we haven't talked about yet?

Is there anything else we haven't talked about that you'd like people to know about how working at [cooperative] has impacted client care, aides' jobs, or aides' lives in general?

Closing Script

This has been incredibly informative, and I really appreciate you taking out the time to discuss this with me. Thanks again for your time and insights.

© 2025 Gusoff GM et al. *JAMA Network Open*.
