# Supplementary material for: Perceived Contributors to Job Quality and Retention at Home Care Cooperatives
Source: JAMA Netw Open. 2025 Apr 7;8(4):e254457. doi: 10.1001/jamanetworkopen.2025.4457 (PMC11976488; doi:10.1001/jamanetworkopen.2025.4457)
Supplement: Supplement 2. — Data Sharing Statement [file jamanetwopen-e254457-s002.pdf]

Data Sharing Statement

Gusoff. Perceived Factors Associated With Higher Job Quality and Lower Turnover at Home Care Cooperatives. *JAMA Netw Open*. Published April 07, 2025. doi:10.1001/jamanetworkopen.2025.4457

Data
Data available: No
